# Post-Stroke Status Epilepticus: Time of Occurrence May Be the Difference?

**DOI:** 10.3390/jcm12030769

**Published:** 2023-01-18

**Authors:** Annacarmen Nilo, Giada Pauletto, Simone Lorenzut, Giovanni Merlino, Lorenzo Verriello, Francesco Janes, Francesco Bax, Gian Luigi Gigli, Mariarosaria Valente

**Affiliations:** 1 Clinical Neurology Unit, Department of Neurosciences, Santa Maria della Misericordia University Hospital, 33100 Udine, Italy; 2 Neurology Unit, Department of Neurosciences, Santa Maria della Misericordia University Hospital, 33100 Udine, Italy; 3 Department of Medicine (DAME), University of Udine Medical School, 33100 Udine, Italy

**Keywords:** status epilepticus, stroke, outcome, post-stroke epilepsy, seizure recurrence

## Abstract

(1) Background: Stroke is one of the most frequent causes of status epilepticus (SE) in adults. Patients with stroke and SE have poorer prognosis than those with stroke alone. We described characteristics and prognosis of early- and late-onset post-stroke SE (PSSE). (2) Methods: We retrospectively analyzed consecutive stroke patients who experienced a first SE between August 2012 and April 2021, comparing clinical characteristics, stroke, and SE features between early- versus late-onset SE in relation to patients’ outcome. (3) Results: Forty stroke patients experienced PSSE. Fourteen developed an early-onset SE (35%) and twenty-six a late-onset SE (65%). Early-onset SE patients had a slightly higher NIHSS score at admission (6.9 vs. 6.0; *p* = 0.05). Early-onset SE was more severe than late-onset, according to STESS (Status Epilepticus Severity Score) (3.5 vs. 2.8; *p* = 0.05) and EMSE (Epidemiology-based Mortality score in Status Epilepticus) score (97.0 vs. 69.5; *p* = 0.04); furthermore, it had a significant impact on disability at 3-month and 1-year follow-up (*p* = 0.03 and *p* = 0.02). SE recurrence and seizures relapse were observed mainly in cases of late-onset SE. (4) Conclusions: Early-onset SE seems to be associated with higher disability in short- and long-term follow-up as possible expression of severe acute brain damage.

## 1. Introduction

Status epilepticus (SE) is a life-threatening condition, especially in elderly and critically ill patients. Acute stroke has been reported to cause up to 22% of all cases of SE in adults [1]. Considering the overall stroke population, SE occurs in 1.5%, a proportion which increases to more than 10% among patients with stroke-related seizures [1,2]. Although even isolated seizures can negatively affect post-stroke outcome and quality of life and increase hospital costs, patients with stroke and SE have poorer prognosis than those with stroke alone [3,4,5].

SE is seldom described in studies reporting post-stroke seizures; only a few reports have focused exclusively on post-stroke SE (PSSE) [3,5,6,7]. Stroke severity, extensive cortical involvement, hemorrhagic transformation, alcohol abuse, sodium imbalance, and involvement of temporal regions are the most relevant risk factors for post-stroke seizures and also for PSSE [5,7,8,9,10,11].

The incidence of SE is higher in the first week after stroke. Timing of PSSE, stroke severity, and SE duration negatively influence long-term outcome and mortality [12].

Treatment of acute stroke has changed in the last decades, and the widespread access to reperfusion therapies has deeply modified stroke management. Thus, semi-intensive cares in stroke units have allowed a prompt recognition of stroke complications such as PSSE and a consequent more aggressive treatment.

The aim of this study is to describe characteristics and prognosis of early- and late-onset PSSE regarding patients’ disability, long-term risk of seizures relapse, and SE recurrence in a monocentric cohort of subjects who were admitted and treated in a semi-intensive care stroke unit setting.

## 2. Materials and Methods

### 2.1. Study Design and Data Collection

In this retrospective study, we included patients with acute ischemic and hemorrhagic stroke who were admitted to our stroke unit and developed a first episode of SE between August 2012 and April 2021.

Acute stroke was defined as presence of neurological signs and symptoms that were attributable to a specific vascular region, lasting more than 30 min, with other possible causes being excluded by neuroimaging performed within 4.5 h from the onset. Patients with unruptured arterial malformations and subarachnoid or subdural hemorrhages were excluded.

Based on the International League Against Epilepsy (ILAE) definition [13], status epilepticus was defined as the occurrence of a prolonged seizure (at least 5 min for tonic-clonic seizure and 10 min for focal seizure with impaired consciousness) or a series of seizures with incomplete return to baseline. Non-convulsive status epilepticus was also defined electroencephalographically according to the Salzburg criteria [14]. SE was established as early-onset if it occurred within 7 days from stroke and as late-onset when appearing after 7 days. According to the SE classification of the ILAE [13], episodes with prominent motor symptoms, including generalized convulsive SE, myoclonic SE, and focal motor SE, were classified as convulsive SE (CSE), whereas episodes without prominent motor symptoms, including non-convulsive SE in coma and focal SE with impaired consciousness, were considered as non-convulsive SE (NCSE).

Electroencephalograms (EEGs), performed according to the 10/20 International System, were recorded if patients presented symptoms consistent with CSE and/or NCSE. EEG recordings were carried out until SE resolution. Two neurophysiologists expert on epilepsy (A.N. and G.P.) reviewed each EEG retrospectively.

Clinical information was retrieved from medical records.

At baseline, we collected the following data: age, gender, vascular risk factors (hypertension, diabetes mellitus, dyslipidemia, smoke, and alcohol abuse), baseline functional status using the modified Rankin scale (mRS), stroke type (ischemic and hemorrhagic), etiology (according to Trial of org 101172 in acute Stroke Treatment (TOAST) classification) and localization, and stroke severity (according to the National Institutes of Health Stroke Scale, NIHSS). Then, clinical outcome in terms of disability (according to the mRS) and mortality was evaluated at discharge, 3-month, and 1-year follow-up. The mRS after discharge was recorded at the patients’ routine clinical visit or through telephone interview with patients or their immediate caregivers. Patients with a mRS score between 3 and 5 were considered functionally dependent.

The features of the first episode of PSSE included time from stroke onset to SE, SE type and duration, and acute treatment (type and number of anti-seizure medications, ASMs). The Status Epilepticus Severity Score (STESS) [15] and the Epidemiology-based Mortality score in Status Epilepticus (EMSE) [16] were calculated for each patient. Refractoriness was established when SE did not resolve after at least two ASMs used at appropriate doses.

The local Ethics Committee (Comitato Etico Unico Regionale del Friuli Venezia Giulia) approved this investigation (Ref. No CEUR-2020-Os-173).

### 2.2. Statistical Analysis

All data were collected in an ad hoc created database (Excel 2022, Microsoft Corp., Redmond, WA, USA). The average imputation method and the last observation carried forward method were used to handle missing values (<5% of missing data). Continuous variables were summarized by descriptive statistics expressed as mean, median, interquartile interval, and range and categorical variables by absolute frequencies and percentages. The *t*-test or Mann–Whitney U-test, as appropriate, was used to compare continuous variables between groups. For categorical variables, cross-tabulations were generated, and a chi-square or Fisher’s exact test was used to compare distributions, as appropriate. The impact of SE type on clinical outcome in term of disability, evaluated as mRS score (dichotomized in score ≤ 2 or >2) at discharge, at 3 months, and at 1 year, was evaluated by a univariate binary logistic regression. Then, a multivariable logistic regression model was built to estimate the odds ratio (OR) of mRS > 2 at discharge, at 3 months, and at 1 year. Model building was achieved via a purposeful method and clinical plausibility. A Cox proportional hazard regression was used to evaluate risk factors affecting survival in patients with SE, and proportionality assumption was tested by plotting Schoenfeld residuals over time.

Statistical significance was set at 0.05.

All statistical analyses were performed using SPSS version 25.0 (IBM Corporation, Chicago, IL, USA) and R Software for survival analysis (version 4.2.0).

## 3. Results

### 3.1. Demographic and Clinical Characteristics

A total of 40 out of 3998 stroke patients experienced a first episode of PSSE (1%) between August 2012 and April 2021. Ninety-five patients of the whole cohort presented at least one acute symptomatic seizure, among these 12 patients developed an SE (7 an early-onset PSSE and 5 a late-onset PSSE).

The baseline characteristics of total stroke patients are summarized in Table 1.

Patients included were predominantly women (24/40, 60%), with a median age of 77 years (range 18–91). The median baseline mRS was 1.0 (IQR 2). Twenty-one patients (52.5%) died during the follow-up period, nine of them (42.8%) due to a cause other than PSSE. Twelve patients (30%) died during the acute phase as consequence of SE.

Demographic characteristics, comorbidities, stroke features, and mRS and NIHSS score are reported in details in Table 2.

### 3.2. PSSE Characteristics

Based on the ILAE SE classification, 17 (42.5%) patients presented CSE, while 23 (57.7%) subjects experienced NCSE. Half of all PSSE (*n* = 20) were refractory, and 12 of them (30%) required therapeutic coma to control SE (5 with late-onset PSSE and 7 with early-onset PSSE), with a median of intensive care unit (ICU) stay of 3.8 days (range: 2–7). A median of 2.0 ASMs (IQR 1; range: 1–5) were used for SE treatment. Levetiracetam and lacosamide were the most frequent used ASMs (85% and 50%, respectively), while the most used anesthetic was propofol (11/12 patients).

Fourteen patients experienced an early-onset SE (35%) with an equally representation of CSE and NCSE. Twenty-six subjects developed a late-onset SE with a median latency of 14 months from stroke onset (range: 2–30 months). Detailed clinical presentation of PSSE is described in Table 3.

Comparing early-onset versus late-onset SE, there were no significant differences in gender, type of stroke (ischemic vs. hemorrhagic), and chronic vascular comorbidities (hypertension, diabetes, and dyslipidemia) between the two groups.

The age of the two groups was not significantly different (75.50 years for early- vs. 77.50 years for late-onset SE). The early-onset SE subgroup showed a slightly higher NIHSS score (6.9 vs. 6.0; *p* = 0.05). Furthermore, early-onset SE was more severe than the late-onset one, according to STESS (3.5 vs. 2.8, respectively; *p* = 0.05) and EMSE score (97.0 vs. 69.5, respectively; *p* = 0.04). SE duration and number of ASMs did not significantly differ between the two groups (Table 3).

The mortality was comparable between early-onset and late-onset SE patients; only age showed a borderline correlation with mortality (Table 4). Early-onset SE had a significant impact on disability at discharge, at 3-month, and at 1-year follow-up (*p* = 0.04, *p* = 0.03 and *p* = 0.02, respectively), at univariate analysis. The multivariable logistic analysis confirmed the association between disability and SE severity, evaluated as STESS score, but only at discharge (Table 5).

Other parameters, such as age, sex, stroke severity, SE type, reperfusion therapies, SE duration, and ICU stay, did not significantly influence patients’ outcomes.

SE recurrence and seizures relapse were observed mainly in the case of late-onset SE: 7 patients with late-onset SE had recurrence of SE with a median interval of 12 months from the first episode, while 18 subjects (69.2%) showed seizures recurrence. No patients with early-onset SE experienced other SE, and just one presented a single seizure relapse.

## 4. Discussion

Status epilepticus is a serious complication of acute stroke. Reports in the literature regarding post-stroke SE vary according to study design and availability of EEG in the clinical setting [11]. Furthermore, not all studies on PSSE focused on patients admitted to stroke units.

Our study takes into consideration only patients admitted to stroke unit for possible reperfusion therapy; thus, they had higher and more standardized care with continuous monitoring of vital parameters and neurological status. These aspects allowed a prompt recognition of SE and, consequently, early EEG recording and treatment.

We focused on the possible differences between patients with early- and late-onset PSSE to better define short- and long-term management and their prognosis. As far as we know, this topic has not been widely analyzed in previous reports [5,12,17].

Labovitz and colleagues [1], in their population study, found that lesion location and stroke subtype (deep infarct, lobar infarct, deep intracerebral hemorrhage, lobar intracerebral hemorrhage (ICH), and subarachnoid hemorrhage) were strong determinants of early seizures and early SE risk [1]. Bateman et al. [7] analyzed the occurrence of generalized CSE in acute ischemic stroke (AIS) and ICH using a large discharge database [7]. Female sex, African American race, renal disease, alcohol abuse, sodium imbalance, and hemorrhagic transformation were associated with higher rates of generalized CSE within the AIS cohort, while African American and Hispanic race, renal disease, coagulopathy, brain tumor, alcohol abuse, and sodium imbalance were associated with higher rates of generalized CSE for the ICH cohort [7]. The metanalysis performed by Wang et al. confirmed the same risk factors for PSSE [18].

In our study, comparing early- and late-onset SE, demographic features and stroke type did not differ between the two groups. Early-onset SE was more likely to occur in presence of hemorrhagic frontal stroke (both cortical hemorrhage and hemorrhagic evolution of ischemic stroke), while late-onset SE seemed to be slightly more frequent in case of ischemic temporal stroke, without a statistical level of significance. These data are consistent with the study of Tomari and colleagues [19] and also of Belcastro et al. [11]: the first one reported a higher incidence of post-stroke NCSE in case of frontal stroke, while the latter observed a predominant correlation with temporal lobe stroke. Study design may explain the differences: in fact, we considered all cases of PSSE and not only subjects with NCSE.

We confirmed the previous observations that patients with a higher NIHSS had an increased risk of developing acute symptomatic seizures and early-onset SE [5,12,20]. In particular, a recent retrospective study showed that NIHSS score after 24 h was the strongest predictive factor for the occurrence of acute symptomatic seizures as consequence of a larger volume of damaged brain tissue and concomitant cortical involvement [20]. Moreover, we observed globally a high mortality rate in our cohort regardless of the timing of PSSE occurrence. However, patients with early-onset SE seemed to present a more severe disability at short-term (discharge and 3-months) and long-term (1-year) follow-up. Our data are consistent with other studies that pointed out that prognosis of patients with PSSE is generally poorer [5,7,21]. Santamarina et al. [5], in their large national study on PSSE, demonstrated that early time of onset (<72 h), baseline mSTESS, and stroke lesion extension are independent factors predicting functional decline at discharge and at 3 months [5].

In our study, the poor prognosis is mainly due to disability that was assessed in a longer follow-up (1 year). Although patients with early-onset SE had a slightly higher NIHSS score, the stroke severity did not statistically differ between the two groups and did not seem to affect patients’ outcome. Disability could depend not only on the stroke severity but also on the occurrence of SE; in fact, the persistence of epileptic activity contributes to the neurotoxic effects that may result in a worse outcome [22,23]. Our results showed, at univariate analysis, a correlation between PSSE type (in particular early-onset PSSE) and functional disability at discharge, at 3 months, and at 1 year. The multivariable logistic regression model confirmed only a correlation between STESS score and disability at discharge, supporting the possible aggravating role of SE severity on functional outcome. Nevertheless, we should consider that mRS assesses predominantly motor impairment, and we did not evaluate other scales measuring quality of life or cognition that may be more affected by the occurrence of SE, in particular in the late-phase.

Post-stroke epilepsy and SE recurrence do not seem to be related with early-onset SE, instead being more frequent in the case of late-onset SE. Our data are different from those reported by Velioglu et al. [21] and Abraira et al. [12], who observed that early-onset SE was associated with a higher risk for SE recurrence. However, in both studies, they did not compare directly early-onset SE versus late-onset SE, but they considered patients with PSSE versus patients without PSSE or patients with versus without post-stroke seizures. The major risk of SE recurrence and seizures relapse in late-onset SE is consistent with the establishment of epileptogenic mechanisms represented by altered synaptic plasticity, reverberant circuit, and neuronal sprouting, while early-onset SE mainly represents the effect of acute brain sufferance and neuronal excitotoxicity [24]. Animal models are used to describe acute and chronic effects of different brain injuries and evaluate the role of neuronal hyperexcitability, inflammation, and neurodegeneration on epileptogenesis [25]. Following brain insults, such as stroke, a cascade of morphological and functional changes takes place over months or even years before spontaneous epileptic firing occurs. This sort of “silent period” may represent a therapeutic window to interrupt or prevent epileptogenic processes [25,26]. Therefore, it is possible that early-onset SE is treated promptly and more aggressively from the beginning, determining a possible delay and smoothing of epileptogenic processes. The degree of acute brain sufferance is expressed by a higher STESS reported by Santamarina et al. [5] and confirmed by our study.

## 5. Limitations

The present work has some limitations. The most important is due to the small sample size. This aspect may interfere with the power of our statistical results, in particular regarding multivariable analysis. In addition, it is a retrospective single-center study, and it carries all the limits intrinsic to this study design. Furthermore, it comes from a single center: a guarantee of homogeneity but also a risk of unintended biases. Finally, since it covers ten years, the expertise of the stroke team and the EEG protocol in acute stroke patients have changed; thus, it is possible that some cases, especially in the first years of observation, may have been missed, particularly in patients with NCSE whose diagnoses necessarily require an EEG recording.

## 6. Conclusions

Status epilepticus can affect mortality and disability in stroke patients. In particular, our results showed a possible association between early-onset SE and higher disability in short- and long-term follow-up as an expression of a more severe acute brain damage. Further, larger studies are needed to confirm our results and to assess the best management of stroke patients to detect SE and avoid the subsequent development of epileptogenic mechanisms.

## Figures and Tables

**Table 1:** Baseline characteristics of whole stroke population.

	*n* = 3998
Age, years; median (IQR, min–max)	74.0 (16, 18–99)
Gender (female); *n* (%)	1688 (42.2%)
Alcohol abuse; *n* (%)	799 (20%)
Smoke; *n* (%)	1450 (36.3%)
Vascular risk factors; *n* (%)	
Hypertension	2799 (70%)
Diabetes mellitus	1200 (30%)
Dyslipidemia	1198 (29.9%)
Baseline mRS; median (IQR, min–max)	0.7 (1, 0–5)
Stroke type; *n* (%)	
Ischemic	3312 (82.8%)
Hemorrhagic	686 (17.2%)
NIHSS score; median (IQR, min–max)	7.5 (8, 1–27)

Abbreviations: IQR, interquartile range; mRS, modified Rankin scale; NIHSS, National Institutes of Health Stroke Scale.

**Table 2:** Demographics characteristics, comorbidities, and stroke clinical features of study populations.

	Early-Onset PSSE (*n* = 14)	Late-Onset PSSE (*n* = 26)	*p*-Value
**DEMOGRAPHIC and CLINICAL FEATURES**			
Age, years; median (IQR, min–max)	75.50 (14, 57–89)	77.50 (18, 18–91)	0.09
Gender (female); *n* (%)	9 (64.3%)	15 (57.7%)	0.48
Alcohol abuse; *n* (%)	2 (14.3%)	6 (23.1%)	0.41
Smoke; *n* (%)	4 (28.6%)	2 (7.7%)	0.09
Vascular risk factors; *n* (%)			
Hypertension	11 (78.6%)	18 (69.2%)	0.40
Diabetes mellitus	5 (35.7%)	3 (11.5%)	0.08
Dyslipidemia	5 (35.7%)	7 (26.9%)	0.41
Baseline mRS; median (IQR, min–max)	1.0 (2, 0–4)	1.0 (1, 0–4)	0.85
**STROKE FEATURES**			
Stroke type (*n*, %)			
Ischemic/hemorrhagic	7/7 (50/50%)	15/11 (57.7/42.3%)	0.45
Ischemic etiology (TOAST); *n* (%)			-
Cardioembolic	0 (0%)	7 (46.7%)	
Atherothrombotic	0 (0%)	7 (46.7%)	
Undetermined dissection	7 (100%)	1 (6.7%)	
Hemorrhagic characteristics; *n* (%)			-
Cortical/subcortical	7/0 (100/0%)	9/2 (81.8/18.2%)	
Intraventricular	0 (0%)	0 (0%)	
Primary/secondary form	6/1 (85.7/14.3%)	11/0 (100/0%)	
Lesion localization; *n* (%)			0.53
Frontal	6 (42.9%)	7 (26.9%)	
Temporal	4 (28.6%)	9 (34.6%)	
Parietal	3 (21.4%)	4 (15.4%)	
Occipital	1 (7.1%)	2 (7.7%)	
Other	0 (0%)	4 (15.4%)	
NIHSS score; median (IQR, min–max)	6.9 (7, 0–18)	6.0 (6, 1–21)	*0.05*
NIHSS score > 5; *n* (%)	11 (78.6%)	12 (46.1%)	

Borderline values are in italics. Abbreviations: PSSE, post-stroke status epilepticus; IQR, interquartile range; mRS, modified Rankin scale; NIHSS, National Institutes of Health Stroke Scale; TOAST, Trial of org 101172 in acute Stroke Treatment.

**Table 3:** Main characteristics of post-stroke status epilepticus.

	Early-Onset PSSE (*n* = 14)	Late-Onset PSSE (*n* = 26)	*p*-Value
PSSE type			
CSE/NCSE; *n* (%)	7/7 (50/50%)	10/16 (38.5/63.5%)	0.35
Level of consciousness			
(stupor or coma); *n* (%)	7 (50%)	9 (34.6%)	0.27
GCS score at SE onset; *n* (%)			0.59
≥14	2 (14.3%)	7 (26.9%)	
9–13	6 (42.9%)	8 (30.9%)	
≤8	6 (42.9%)	11 (42.3%)	
SE duration (hours); median (IQR, min–max)	13.5 (10, 0.5–48)	12.1 (15, 0.5–48)	0.16
Refractory PSSE; *n* (%)	9 (64.3%)	11 (42.3%)	0.71
Number of ASMs; median (IQR, min–max)	2.0 (1, 1–4)	1.5 (1, 1–5)	0.53
Type of ASMs; *n* (%)			-
Levetiracetam	14 (100%)	20 (76.9%)	
Lacosamide	7 (50%)	13 (50%)	
Valproic acid	3 (21.4%)	3 (11.5%)	
Phenytoin	2 (14.3%)	2 (7.7%)	
Brivaracetam	2 (14.3%)	1 (3.8%)	
STESS score; median (IQR, min–max)	3.5 (2, 1–6)	2.8 (2, 0–6)	*0.05*
≤2	2 (14.3%)	11 (42.3%)	
>2	12 (85.7%)	15 (57.7%)	
EMSE score; median (IQR, min–max)	97.0 (42, 54–155)	69.5 (68, 12–157)	**0.04**
<64	2 (14.3%)	12 (85.7%)	
≥64	12 (46.2%)	14 (53.8%)	
mRS at discharge; median (IQR, min–max)	3.0 (3, 1–5)	2.0 (2, 1–6)	**0.04**
mRS at 3 months; median (IQR, min–max)	2.5 (2, 2–4)	2.0 (2, 1–4)	**0.03**
mRS at 1 year; median (IQR, min–max)	4.0 (3, 1–6)	2.0 (4, 1–5)	**0.02**
Death; *n* (%)	8 (57.1%)	13 (50%)	0.52

Borderline values are in italics. Significant values are in bold. Abbreviations: ASMs, anti-seizure medications; CSE, convulsive status epilepticus; EMSE, Epidemiology-based Mortality score in Status Epilepticus; GCS, Glasgow Coma Scale; IQR, interquartile range; mRS, modified Rankin scale; NCSE, non-convulsive status epilepticus; PSSE, post-stroke status epilepticus; STESS, Status Epilepticus Severity Score.

**Table 4:** Cox Proportional Hazard Regression Model.

Mortality			
	HR	95% CI	*p*-Value
Age	1.05	1.00–1.11	*0.05*
Sex	1.74	0.52–5.75	0.36
NIHSS score	0.78	0.26–2.30	0.65
Stroke type	1.74	0.52–5.83	0.37
PSSE type *	0.68	0.25–1.89	0.46
STESS score	2.01	0.56–7.25	0.28
EMSE score	0.87	0.24–3.09	0.82

* Early-onset vs. late-onset SE. Borderline values are in italics. Abbreviations: CI, confidence interval; EMSE, Epidemiology-based Mortality score in Status Epilepticus; HR, hazard ratio; NIHSS, National Institutes of Health Stroke Scale; PSSE, post-stroke status epilepticus; STESS, Status Epilepticus Severity Score.

**Table 5:** Multivariable logistic regression model (adjusted OR and 95% CI for mRS > 2 at discharge, at 3 months, and at 1 year, *n* = 40).

	mRS at Discharge			mRS at 3 Months			mRS at 1 Year		
	Beta	Adj OR (95% CI)	*p*-Value	Beta	Adj OR (95% CI)	*p*-Value	Beta	Adj OR (95% CI)	*p*-Value
Age	−0.02	0.97 (0.91–1.04)	0.5	0.01	1.01 (0.95–1.08)	0.7	−0.02	0.97 (0.91–1.04)	0.5
Sex	0.39	1.48 (0.24–9.14)	0.6	−0.04	0.65 (0.11–3.90)	0.6	−2.17	0.11 (1.33–2.47)	0.1
NIHSS score	0.06	1.06 (0.19–5.73)	0.9	0.15	1.17 (0.24–5.77)	0.8	1.39	4.01 (0.26–61.43)	0.3
Stroke type	1.57	4.79 (0.62–36.9)	0.1	1.10	3.01 (0.52–17.13)	0.2	−22.4	1.81 (0.33–2.47)	0.9
PSSE type *	−0.66	0.51 (0.08–3.35)	0.5	−0.18	0.83 (0.15–4.45)	0.8	−0.84	0.43 (0.03–5.27)	0.5
STESS score	0.97	2.65 (1.07–6.54)	**0.02**	0.53	1.71 (0.88–3.33)	0.1	−2.32	0.79 (0.01–4.56)	0.2
EMSE score	−0.19	0.82 (0.11–5.77)	0.8	0.42	1.53 (0.22–10.50)	0.6	−0.17	0.84 (0.06–12.06)	0.9

* Early-onset vs. late-onset SE. Significant values are in bold. Abbreviations: Adj, adjusted; CI, confidence interval; EMSE, Epidemiology-based Mortality score in Status Epilepticus; mRS, modified Rankin Scale; NCSE, non-convulsive status epilepticus; NIHSS, National Institutes of Health Stroke Scale; OR, odds ratios; PSSE, post-stroke status epilepticus; SE, status epilepticus, STESS, Status Epilepticus Severity Score.

## Data Availability

Data Access, Responsibility and Analysis: All authors had full access to all the data in the study and take responsibility for the integrity of the data and the accuracy of the data analysis.
